# Detection of a streptomycin-resistant *Mycobacterium bovis* strain through antitubercular drug susceptibility testing of Tunisian *Mycobacterium tuberculosis* complex isolates from cattle

**DOI:** 10.1186/s12917-018-1623-9

**Published:** 2018-09-29

**Authors:** Saif Eddine Djemal, Cristina Camperio, Federica Armas, Mariam Siala, Salma Smaoui, Feriele Messadi-Akrout, Radhouane Gdoura, Cinzia Marianelli

**Affiliations:** 1 Department of Life Sciences, Research Laboratory of Environmental Toxicology-Microbiology and Health (LR17ES06), Faculty of Sciences, University of Sfax, Sfax, Tunisia; 2 Department of Food Safety, Nutrition and Veterinary Public Health, Istituto Superiore di Sanità, Viale Regina Elena 299, 00161 Rome, Italy; 3 Department of Biology, Preparatory Institute for Engineering Studies, University of Sfax, Sfax, Tunisia; 4 Department of Microbiology, Regional Hygiene Care Mycobacteriology Laboratory, Hedi-Chaker University Hospital, Sfax, Tunisia; 5 Department of Biology B, Faculty of Pharmacy, University of Monastir, Monastir, Tunisia; 6 Department of Microbiology, National Reference Laboratory of Mycobacteria, Research Unit (UR12SP18), A. Mami University Hospital of Pneumology, Ariana, Tunisia

**Keywords:** Bovine tuberculosis, *Mycobacterium tuberculosis* complex, Streptomycin resistance, Resazurin microtitre assay, d-REMA

## Abstract

**Background:**

A rising isolation trend of drug-resistant *M. bovis* from human clinical cases is documented in the literature. Here we assessed *Mycobacterium tuberculosis* complex isolates from cattle for drug susceptibility by the gold standard agar proportion method and a simplified resazurin microtitre assay (d-REMA). A total of 38 *M. tuberculosis* complex strains, including *M. bovis* (*n* = 36) and *M. caprae* (*n* = 2) isolates, from cattle in Tunisia were tested against isoniazid, rifampin, streptomycin, ethambutol, kanamycin and pyrazinamide.

**Results:**

*M. caprae* isolates were found to be susceptible to all test drugs. All *M. bovis* strains were resistant to pyrazinamide, as expected. In addition, one *M. bovis* isolate showed high-level resistance to streptomycin (MIC > 500.0 μg/ml). Concordant results with the two methods were found. The most common target genes associated with streptomycin resistance, namely the *rrs*, *rpsL* and *gidB* genes, were DNA sequenced. A non-synonymous mutation at codon 43 (K43R) was found in the *rpsL* gene. To the best of our knowledge, this is the first report describing the isolation of a streptomycin-resistant *M. bovis* isolate from animal origin.

**Conclusions:**

Antitubercular drug susceptibility testing of *M. bovis* isolates from animals should be performed in settings where bTB is endemic in order to estimate the magnitude of the risk of drug-resistant tuberculosis transmission to humans.

## Background

The World Organization for Animal Health (OIE) has recognized bovine tuberculosis (bTB) as an important animal disease and zoonosis [1]. bTB causes significant economic losses to farmers due to livestock deaths, reduced productivity and restrictions for trading animals.

The main causal agents of bTB are *Mycobacterium bovis* and, to a lesser extent, *Mycobacterium caprae*, both members of the *Mycobacterium tuberculosis* complex. These pathogens may also cause tuberculosis in humans (hereafter referred to as zoonotic tuberculosis, zTB) although the true incidence of this disease in human beings is unknown [2–4]. An extensive meta-analysis found the proportion of zTB to be ≤1.4% in countries outside Africa and 2.8% on average in African countries [3]. The vast majority of cases were due to *M. bovis* and the contribution of cases due to *M. caprae* was not quantified. Substantial evidence suggests that zTB might be underestimated because of two major issues hindering understanding of the true burden of this disease: first, the absence of systematic surveillance for *M. bovis* and *M. caprae* as cause of tuberculosis in humans in all low-income and high tuberculosis burden countries where bTB is endemic; and second, the inability of laboratory procedures most commonly used to diagnose human tuberculosis to identify and differentiate these pathogens from *M. tuberculosis*, with the result that all cases may be assumed to be caused by *M. tuberculosis* [5].

Animal test-and-slaughter schemes have successfully reduced the prevalence of bTB in most industrialized countries. The situation is profoundly different in unindustrialized countries where the WHO, in conjunction with FAO and OIE, has classified bTB as a neglected zoonosis. In South Africa, as in other regions in Africa, the lack of bTB control programmes [6] makes communities with high HIV/AIDS infection rates and those living in close contact with infected animals or animal products more vulnerable to zTB [7, 8].

*M. bovis* has one of the broadest host ranges of any known zoonotic pathogen and is globally distributed [9]. The phylogenomic analysis of *M. bovis* genomes has recently revealed large-scale polymorphisms, which may contribute to the differential adaptability of the pathogen [10]. *M. bovis* is naturally resistant to pyrazinamide (PZA) [11], which is one of the first-line antibiotics used to treat tuberculosis in humans. The lack of prompt identification of *M. bovis* human cases may result in improper treatments and have lethal consequences [12]. Several studies have documented additional drug resistances in human *M. bovis* isolates over the last two decades, such as toward isoniazid (INH) [13, 14], streptomycin (STR) [15] or multiple drugs [16–19].

*M. caprae*, on the other hand, is evolutionarily older than *M. bovis* and accounts for a smaller burden of zTB. Moreover, it is not globally distributed but primarily restricted to European countries [3, 20, 21]. Its resistance against first-line drugs is rarely documented [22]. Nevertheless, *M. caprae* has received more attention in recent years, particularly due to the increasing number of *M. caprae* outbreaks in wild or domestic animals which pose a threat to human health [21].

While mycobacteria isolated from human cases are generally assessed for drug susceptibility, studies on antitubercular drug susceptibility testing of *M. bovis* and *M. caprae* isolated from animals are limited [23–29]. So far, *M. bovis* isolates from cattle resistant to INH and rifampin (RIF) have been documented in Italy [23] and Brazil [29], to the best of our knowledge. Monitoring of antitubercular drug resistance of *M. bovis* isolated from animals may thus contribute to reducing the risk of drug-resistant *M. bovis* transmission from animals to humans and among human beings.

In Tunisia, bTB is enzootic and the consumption of raw milk and unpasteurized dairy products is common. A previous study demonstrated that raw milk consumers are at high risk of being infected with *M. bovis* [30]. Numerous clinical cases of human extrapulmonary tuberculosis due to *M. bovis* have been recently documented in Tunisia [31, 32] and the consumption of unpasteurized dairy products has been indicated as the most likely source of transmission [33]. Implementation of effective and comprehensive strategies to control bTB and to prevent zTB are therefore of primary importance in the country.

In this study we assessed drug susceptibility of *M. bovis* and *M. caprae* isolates from cattle in Tunisia towards six antitubercular drugs – PZA, INH, RIF, ethambutol (EMB), STR and kanamycin (KAN) – by both the gold standard agar proportion method and the simplified resazurin microtitre assay (REMA), the dichotomous REMA (d-REMA) recently proposed by Marianelli and colleagues [27].

## Methods

All experimental procedures here described were carried out at the Department of Food Safety, Nutrition and Public Animal Health of the Istituto Superiore di Sanità (ISS), (Italy).

### *M. tuberculosis* complex strains

A total of 38 *M. tuberculosis* complex strains, including *M. bovis* (*n* = 36) and *M. caprae* (*n* = 2) previously isolated in Tunisia and molecular typed by spoligotyping and MIRU-VNTR analysis [34], were provided by the University of Sfax (Tunisia). The isolates were analysed for susceptibility to INH, RIF, STR, EMB, KAN and PZA at ISS.

Isolates were subcultured in Middlebrook 7H9 medium (Biolife, Italy) with 10% oleic acid-albumin-dextrose-catalase (OADC) enrichment (Becton Dickinson and Company) before being tested. To aid the dispersion of bacterial clumps, 3-mm glass beads were added to the tubes and bacterial suspensions were vigorous vortexed for 15 s. Any remaining large bacterial clumps were allowed to settle. Bacterial suspensions were then adjusted to match a 1.0 McFarland turbidity standard.

All *M. bovis* and *M. caprae* strains were isolated from lymph node and tissue samples showing tuberculosis-compatible lesions. Samples were collected at the abattoir during the *postmortem* inspection, in accordance with national laws.

### d-REMA testing

The d-REMA test, based on Palomino and colleagues [35] and slightly modified by Marianelli and colleagues [27] by testing only two concentrations per drug – the cut-off value for drug resistance, R, and the cut-off value of REMA drug susceptibility, S – was used. The R values were determined in studies where REMA was validated against the gold standard method [35–40]. The test was carried out in triplicate in 96-well plates, as previously described [27]. Briefly, drug solutions were prepared at concentrations of 20 mg/ml (PZA; Sigma-Aldrich, UK) and 2 mg/ml in distilled water (INH, STR, EMB, and KAN; Sigma-Aldrich, UK) or methanol (RIF; Sigma-Aldrich, UK), filter sterilised, and frozen until used.

One hundred microliters of Middlebrook 7H9 medium supplemented with OADC was inoculated into each well. One hundred microliters of each bacterial suspension – previously adjusted to a 1.0 McFarland standard and then diluted 1:20 in the same medium – was then inoculated. The antitubercular drugs were subsequently added at two final concentrations, R and S: 0.1 (S) and 0.25 (R) μg/ml for INH, 0.25 (S) and 0.5 (R) μg/ml for RIF, 0.5 (S) and 1.0 (R) μg/ml for STR, 2.5 (S) and 3.125 μg/ml for EMB, 2.5 (S) and 3.125 (R) μg/ml for KAN and 100.0 (S) and 800.0 (R) μg/ml for PZA.

Plates were covered with lids, placed in a plastic bag and incubated at 37 °C for 7 days. Finally, 30 μl of freshly prepared 0.01% resazurin solution (Acros Organics, USA) was added to each well. The plates were incubated overnight at 37 °C and assessed for colour development. The d-REMA testing was repeated twice for those isolates showing either resistance or ambiguous chromatic change.

The drug-sensitive *M. bovis* ATCC 19210 (used as negative control) and two resistant *M. avium* strains (used as positive controls) – one resistant to EMB and one resistant to both INH and EMB – from the Italian bacteria collection were included in the study.

### Agar proportion method in Middlebrook 7H11 medium

The agar proportion method was performed in Middlebrook 7H11 agar. The test was carried out according to the approved standard (M24A) for Susceptibility Testing of Mycobacteria, Nocardiae, and Other Aerobic Actinomycetes published by the Clinical and Laboratory Standards Institute (CLSI) [41]. Briefly, the turbidity of the inoculum was adjusted to match a 1.0 McFarland standard, and diluted 1:100 and 1:10,000. One hundred microliters of these solutions was then inoculated into 35 mm plates with and without the test drug. The test was carried out in duplicate. The following final critical drug concentrations were used: 0.2 μg/ml for INH; 1.0 μg/ml for RIF; 7.5 μg/ml for EMB; 2.0 μg/ml for STR; 6.0 μg/ml for KAN; and 100 μg/ml for PZA [40, 41]. After 3 weeks of incubation at 37 °C, the number of colony forming units (CFU) growing on the drug-containing medium was compared with those growing on the drug-free medium and expressed as a percentage of the latter. The isolate was considered resistant if the number of colonies on a medium containing an antimicrobial agent, relative to the number observed on a drug-free medium was ≥1%. Negative (*M. bovis* ATCC 19210) and positive (*M. avium* strains) controls were also included in the test. The test was repeated twice for isolates showing resistance.

### Streptomycin MIC determination by REMA

MIC testing was to be performed only in case of drug resistance. Since *M. bovis* growth was observed only in the presence of STR and the control drug PZA (as expected) in both the d-REMA assay and agar proportion method (see Results), MIC testing was carried out only for STR. The test was performed in triplicate according to Palomino and colleagues [35]. Ten dilutions were tested (2.5, 5.0, 10.0, 20.0, 50.0, 100.0, 200.0, 300.0, 400.0 and 500.0 μg/ml). The MIC was defined as the lowest drug concentration that prevented resazurin colour change from blue to pink. The *M. bovis* ATCC control was also tested.

### DNA sequencing

Sequencing, too, was to be performed only in case of drug resistance. Since resistance was observed only in *M. bovis* and towards STR and the control drug PZA (see Results), the most common target genes associated with resistance to STR encoding 16S rRNA (*rrs*), ribosomal protein S12 (*rpsL*) [42] and a 7-methylguanosine methyltransferase (*gidB*) [43], were investigated in the resistant *M. bovis* isolate and in three randomly selected STR-susceptible *M. bovis* isolates..

DNA was extracted from cultures using a commercial kit (InstaGene Matrix; Bio-Rad Laboratories, Italy). The whole *rrs*, *rpsL* and *gidB* genes were PCR amplified and sequenced. The reference sequence accession number (AC) NC_002945 of *M. bovis* AF2122/97 available at NCBI was used to design primers for the PCR amplification and sequencing of the *rrs* gene. The *rpsL* and *gidB* genes were PCR amplified and sequenced according to Feuerriegel and colleagues [44].

PCR products were analysed by 2% agarose gel electrophoresis, stained with GelRed Nucleic Acid Stain (Biotium Inc., Hayward, CA), purified by ExoSAP-IT PCR Product Cleanup (Affymetrix, CA) and sequenced by using PCR and, if required, sequencing primers. Sequences were analysed using the ABI Prism SeqScape Software, version 2.0 (Applied Biosystems, Foster City, CA). All consensus sequences generated were then compared to the published, drug-sensitive *M. bovis* AF2122/97 reference strain, to detect genetic variation. Mutations were confirmed through resequencing. To distinguish silent from missense mutations, amino acid sequences were theoretically deduced. PCR and DNA sequencing primers used, PCR conditions followed and size of the amplicons obtained are listed in Table 1.

**Table 1:** PCR and DNA sequencing primers

Primer	Sequence (5′ → 3′)	PCR conditions^a^			Size (bp)	Reference
		D (s)	A (°C, s)	E (s)		
*rrs*-F	CGT GGC CGT TTG TTT TGT CA	30	55, 30	90	1744	This study
*rrs*-R	AAG TCC GAG TGT TGC CTC AG					
*rrs*-seq1	GAA GAA GCA CCG GCC AAC TA					
*rrs-*seq2	TTG TAC CGG CCA TTG TAG CA					
*rpsL*-F	ATG AGA CGA ATC GAG TTT GAG	30	55, 30	60	632	[44]
*rpsL*-R	GCT CAA GCG CAC CAT AAA CAA					
*gidB*-F	CGC CGA GTC GTT GTG CT	30	55, 30	60	892	[44]
*gidB*-R	AGC CTG GCC CGA CCT TA					

^a^Each PCR assay includes an initial denaturation step at 95 °C for 15 min to fully denature the template, followed by 35 cycles of denaturation, annealing and extension steps. D, denaturation time in s at 95 °C; A, annealing conditions (temperature and time in s); extension time in s

## Results

### Drug susceptibility

**Fig. 1:**
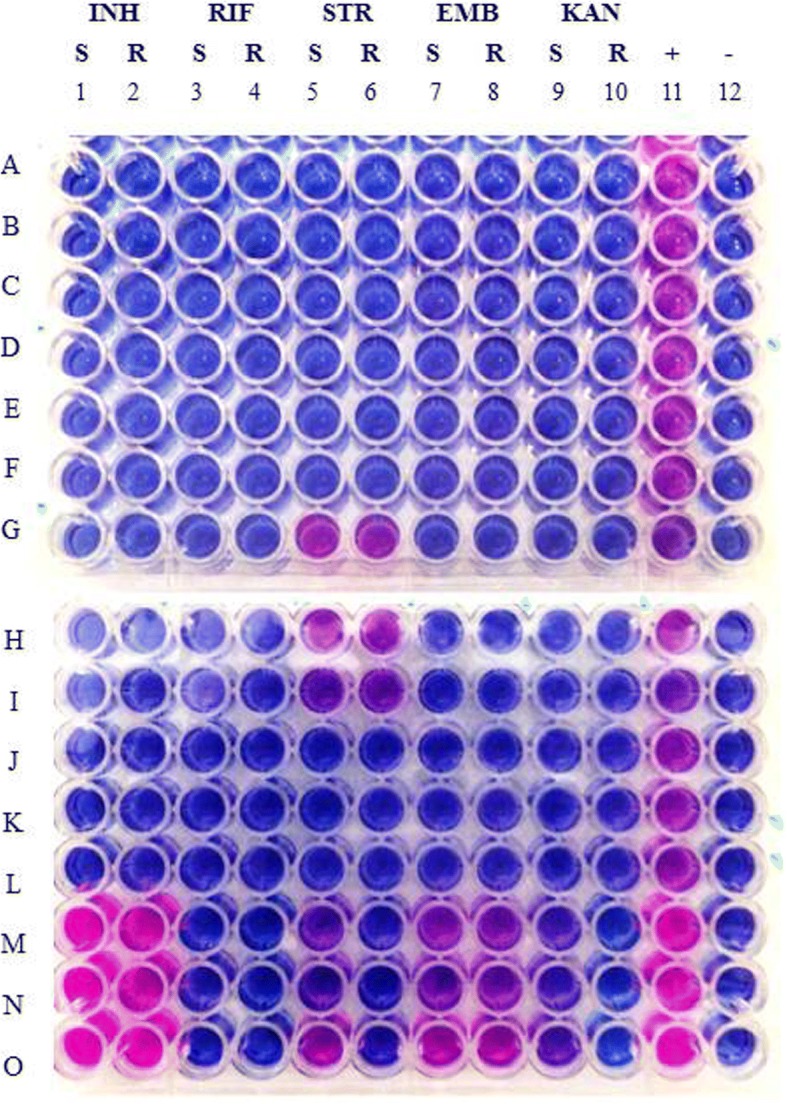
Drug susceptibility profiles. Drug susceptibility to INH, RIF, STR, EMB and KAN by d-REMA. A–C, D–F, G–I: sample triplicates. J–L: *M. bovis* ATCC control in triplicates; M–O: *M. avium* control resistant to both INH and EMB in triplicates. 1–2: 0.1 (S) and 0.25 (R) μg/ml for INH; 3–4: 0.25 (S) and 0.5 (R) μg/ml for RIF; 5–6: 0.5 (S) and 1.0 (R) μg/ml for STR; 7–8: 2.5 (S) and 3.125 (R) μg/ml for EMB: 9–10: 2.5 (S) and 3.125 (R) μg/ml for KAN; (+) positive control containing no drug; (−) negative control containing uninoculated media

*M. bovis* and *M. caprae* isolates, as well as control strains, have been tested here against PZA, INH, RIF, EMB, STR and KAN by both the agar proportion method and d-REMA.

d-REMA results were obtained after 8 days of incubation. All *M. bovis* strains, including the *M. bovis* ATCC control, were resistant to PZA as expected. One out of 36 *M. bovis* isolates showed an additional drug resistance, the STR resistance as shown in Fig. 1, lines G–I. On the other hand, *M. caprae* isolates showed sensitivity against all drugs. The susceptibility of the *M. bovis* ATCC control to all test drugs (Fig. 1, lines J–L) was confirmed, as was the resistance of the two *M. avium* controls towards either INH or INH and ETB (Fig. 1, lines M–O). Results were confirmed through retesting the resistant isolate and controls by both drug susceptibility methods.

After 3 weeks of incubation, d-REMA results were confirmed by the agar proportion method.

**Fig. 2:**
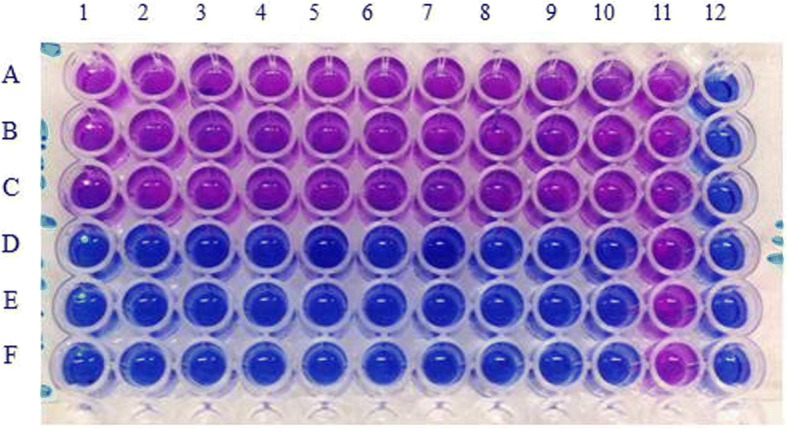
STR MIC results. A–C STR-resistant *M. bovis* isolate in triplicates. D–F: *M. bovis* ATCC control in triplicates. 1–10: 2.5, 5.0, 10.0, 20.0, 50.0, 100.0, 200.0, 300.0, 400.0 and 500.0 μg/ml for STR; (+) positive control containing no drug; (−) negative control containing uninoculated media

The STR-resistant *M. bovis* isolate was subjected to further investigation, including STR MIC determination and DNA sequencing. The isolate showed resistance to all ten test dilutions: the blue-to-mauve colour change occurred at MIC values ranging from 2.5 to 500.0 μg/ml. We were thus unable to determine the STR MIC, as shown in Fig. 2, lines A–C. The *M. bovis* ATCC control, on the other hand, did not grow at any drug concentration tested (Fig. 2, lines D–F).

### DNA sequencing of drug target genes

We then PCR amplified and sequenced the most common target genes associated with resistance to STR, namely the *rrs*, *rpsL* and *gidB* genes, in the resistant *M. bovis* isolate and in three randomly selected STR-susceptible *M. bovis* isolates.

A consensus sequence for each gene was generated and compared to the reference strain *M. bovis* AF2122/97 available in GenBank to detect genetic variation. A non-synonymous mutation – nucleotide substitution AAG → AGG at codon 43 (mutation K43R) – was found only in the *rpsL* gene of the STR-resistant isolate. No mutations were detected in the other two target genes in either of the isolates.

## Discussion

Tunisia is an endemic country for bTB. Despite the implementation of a national bTB control programme, many intensive farms belonging to the state or parastatal sector still do not meet sanitary standards for effective prophylaxis; others, belonging to private owners, also have scarce, if any, veterinary activity [45]. bTB therefore continues to be widespread in this country, mainly in the private sector, which owns more than 70% of the cattle livestock [46]. In Tunisia, the consumption of raw milk and unpasteurized dairy products is common. A previous study has shown that raw milk may spread *M. bovis*, putting consumers of raw milk or derivatives at high risk of being infected [30]. Although the exact contribution of *M. bovis* to the burden of zTB in Tunisia remains unknown, recent studies have indicated *M. bovis* as the main etiological agent of human extrapulmonary tuberculosis [31–33]. In consideration of the above, bTB represents a serious public health problem in Tunisia and effective disease control programmes have to be implemented urgently.

So far, no study has been conducted to estimate the risk of drug or multi-drug resistant bTB transmission to humans in Tunisia where *M. bovis* infection is endemic in livestock. On that account, we assessed at ISS the susceptibility of 36 *M. bovis* and two *M. caprae* isolates from Tunisia to six antibiotic drugs commonly administered to patients with tuberculosis. We detected STR resistance in one *M. bovis* isolate and characterized the nucleotide mutation associated with that resistance.

Drug susceptibility testing is rarely carried out on *M. bovis* and other *Mycobacterium* isolates of animal origin although bTB poses a serious threat to human health particularly in low-income and bTB endemic countries [3, 5]. Multidrug-resistant *M. bovis* outbreaks in humans have occurred, some with serious consequences [16, 18, 47]. *M. bovis* isolates from cattle with drug resistance towards either RIF or INH or towards both drugs have already been documented [23, 29]. Drug susceptibility surveillance of *M. bovis* from animals may therefore contribute to preventing the transmission of multidrug-resistant strains to humans and to controlling possible outbreaks.

Several techniques for testing mycobacterial drug susceptibility are available for *M. tuberculosis* and include the conventional assays [48] and the more rapid colorimetric [49] and molecular methods [50]. The WHO has recently recommended the use of colorimetric assays, which are highly sensitive, specific, rapid and inexpensive methods employing specific reagents to produce a change in colour [51].

Among these, REMA, an indirect method based on the reduction of the coloured dye resazurin added to liquid culture medium on a microtitre plate after exposure of mycobacterial strains to antituberculosis drugs in vitro, was successfully tested on human isolates of *M. tuberculosis* [35, 36, 39, 40]. The REMA plate method proposed by Palomino and colleagues [35] has recently been simplified by Marianelli and colleagues by testing only two concentrations per drug per isolate, the R and S cut-off values, the d-REMA assay [27]. Here, we used both d-REMA and the agar proportion method to assess the bovine isolates for drug susceptibility. We found agreement between the results from the two methods. *M. caprae* isolates were sensitive to all test drugs and *M. bovis* isolates were all PZA resistant, as expected. In addition, one *M. bovis* isolate showed high-level resistance to STR (MIC > 500.0 μg/ml). To our knowledge, this is the first study describing an STR-resistant *M. bovis* isolate of animal origin. We further investigated the STR resistance here observed by sequencing the most common STR target genes, namely the *rrs*, *rpsL* and *gidB* genes. We found a non-synonymous mutation AAG → AGG at codon 43 (K43R) in the *rpsL* gene.

The K43R substitution in the *rpsL* gene is the single most frequent mutation associated with high-level STR resistance in *M. tuberculosis* [42, 52]. It may explain why our *M. bovis* isolate still grew at the highest STR concentration tested (500.0 μg/ml), preventing us from determining the STR MIC. Although the most common target genes associated with resistance to STR have been analysed, we cannot exclude, however, that other mechanisms may be involved. The isolation from human clinical cases of highly STR-resistant *M. tuberculosis* strains carrying the K43R substitution is largely documented in the literature [53–55]. The isolation of STR monoresistant [15] and multidrug-resistant *M. bovis* isolates from humans [16–19] is also described. Drug- and multidrug-resistant tuberculosis is one of the major threats to human medicine. It leads to treatment failures and, in the worst cases, to untreatable infections that cause death.

A recent 15-year laboratory-based surveillance programme conducted in Mexico City on mycobacterial isolates from human clinical samples showed a rising trend of *M. bovis* isolates which caused a higher proportion of pulmonary tuberculosis than previously observed in that area [15]. Additionally, the authors described an increasing rate of primary STR monoresistance in *M. bovis* isolates from humans over time, perhaps as a result of STR usage in cattle [15].

Our results, coupled with the literature, suggest that overuse and misuse of antibiotics in cattle from bTB endemic areas may lead to the development of drug-resistant *M. bovis* strains which, consequently, put humans at risk for primary drug resistant zTB. More effective strategies to reduce antibiotic use in farm animals should therefore be implemented urgently.

## Conclusions

We describe, for the first time, the detection of high-level STR resistance in *M. bovis* from animal origin. Our results suggest that antitubercular drug susceptibility testing of *M. bovis* isolates from animals should be performed in settings where bTB is endemic in order to estimate the magnitude of the risk of drug-resistant tuberculosis transmission to humans.

## Abbreviations

bTB: bovine tuberculosis
CFU: Colony forming units
CLSI: Clinical and Laboratory Standards Institute
d-REMA: dichotomous resazurin microtitre assay
EMB: Ethambutol
FAO: Food and Agriculture Organization
INH: Isoniazid
KAN: Kanamycin
MIC: Minimal inhibitory concentration
NCBI: National Center for Biotechnology Information
OADC: Oleic acid-albumin-dextrose-catalase
OIE: World Organization for Animal Health
PZA: Pyrazinamide
REMA: Resazurin microtitre assay
RIF: Rifampin
STR: Streptomycin
WHO: World Health Organization
zTB: zoonotic tuberculosis
